# Primary mucosal melanomas of the head and neck are characterised by overexpression of the DNA mutating enzyme APOBEC3B

**DOI:** 10.1111/his.14843

**Published:** 2022-12-05

**Authors:** Prokopios P Argyris, Jordan Naumann, Matthew C Jarvis, Peter E Wilkinson, Dan P Ho, Mohammed N Islam, Indraneel Bhattacharyya, Rajaram Gopalakrishnan, Faqian Li, Ioannis G Koutlas, Alessio Giubellino, Reuben S Harris

**Affiliations:** ^1^ Department of Biochemistry, Molecular Biology and Biophysics University of Minnesota Minneapolis MN USA; ^2^ Masonic Cancer Center University of Minnesota Minneapolis MN USA; ^3^ Institute for Molecular Virology University of Minnesota Minneapolis MN USA; ^4^ Center for Genome Engineering University of Minnesota Minneapolis MN USA; ^5^ Howard Hughes Medical Institute University of Minnesota Minneapolis MN USA; ^6^ Division of Oral and Maxillofacial Pathology School of Dentistry, University of Minnesota Minneapolis MN USA; ^7^ Department of Diagnostic and Biological Sciences School of Dentistry, University of Minnesota Minneapolis MN USA; ^8^ Department of Oral and Maxillofacial Diagnostic Sciences University of Florida College of Dentistry Gainesville FL USA; ^9^ Department of Laboratory Medicine and Pathology Medical School, University of Minnesota Minneapolis MN USA

**Keywords:** APOBEC3B, APOBEC3G, DNA cytosine deamination, *HRAS*, *KIT*, *NRAS*, oral mucosal melanoma, sinonasal mucosal melanoma

## Abstract

**Aims:**

Primary head/neck mucosal melanomas (MMs) are rare and exhibit aggressive biologic behaviour and elevated mutational loads. The molecular mechanisms responsible for high genomic instability observed in head/neck MMs remain elusive. The DNA cytosine deaminase APOBEC3B (A3B) constitutes a major endogenous source of mutation in human cancer. A3B‐related mutations are identified through C‐to‐T/−G base substitutions in 5′‐TCA/T motifs. Herein, we present immunohistochemical and genomic data supportive of a role for A3B in head/neck MMs.

**Methods and results:**

A3B protein levels were assessed in oral (*n =* 13) and sinonasal (*n =* 13) melanomas, and oral melanocytic nevi (*n =* 13) by immunohistochemistry using a custom rabbit α‐A3B mAb (5210‐87‐13). Heterogeneous, selective‐to‐diffuse, nuclear only, A3B immunopositivity was observed in 12 of 13 (92.3%) oral melanomas (H‐score range = 9–72, median = 40) and 8 of 13 (62%) sinonasal melanomas (H‐score range = 1–110, median = 24). Two cases negative for A3B showed prominent cytoplasmic staining consistent with A3G. A3B protein levels were significantly higher in oral and sinonasal MMs than intraoral melanocytic nevi (*P* < 0.0001 and *P* = 0.0022, respectively), which were A3B‐negative (H‐score range = 1–8, median = 4). A3B levels, however, did not differ significantly between oral and sinonasal tumours (*P* > 0.99). NGS performed in 10 sinonasal MMs revealed missense *NRAS* mutations in 50% of the studied cases and one each *KIT* and *HRAS* mutations. Publicly available whole‐genome sequencing (WGS) data disclosed that the number of C‐to‐T mutations and APOBEC3 enrichment score were markedly elevated in head/neck MMs (*n* = 2).

**Conclusion:**

The above data strongly indicate a possible role for the mutagenic enzyme A3B in head/neck melanomagenesis, but not benign melanocytic neoplasms.

## Graphical abstract

Nuclear APOBEC3B (A3B) upregulation characterizes head/neck mucosal melanomas, fuels mutational load, and promotes inter‐ and intratumoral heterogeneity.

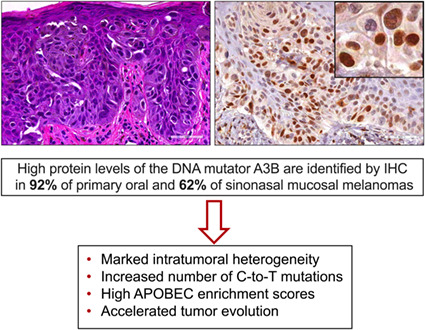

## Introduction

Primary mucosal melanomas (MMs) are exceedingly rare neoplasms and biologically distinct from their cutaneous counterpart.^1^ MMs account for approximately 1.3% of all melanomas and 0.03% of human cancers,^2^ with an estimated 800 cases per year in the US.^3^ Approximately 45–55% of MMs are localised in the head/neck region and arise predominantly in the sinonasal sites (50–80%)^1^, ^3^ and oral cavity (25%).^3^, ^4^ The patient age range is wide, with an incidence peak in the 7th decade of life.^1^ No gender predilection has been reported.^5^

Although UV radiation comprises a major aetiologic factor for cutaneous melanomas, the aetiology of head/neck MMs remains elusive, in part due to the relative rarity of the disease.^6^, ^7^ At the genomic level, primary oral and sinonasal MMs are characterised by a high number of chromosomal structural aberrations and increased mutational burden.^8^, ^9^ Their molecular profile is evidently distinct from the underlying mutations occurring in cutaneous and ocular melanomas.^10^ *KIT* (CD117) mutations are detected in 10–37% of MMs, followed by *NRAS* mutations (15–20%), whereas *BRAF* V600E abnormalities are rare (<6%),^3^, ^11^, ^12^ limiting the use of BRAF inhibitors in these lesions. Combined locoregional and distant metastases develop in 50% of MMs of the head/neck^3^, ^13^ and the overall prognosis is dismal; 5‐year survival is limited to 25–30% with a median survival of 24 months.^14^, ^15^, ^16^

The past decade has enabled a remarkable view of human genomic DNA sequences and, therefore, also of the overall mutation landscape of human cancers. Interestingly, a large fraction of mutations is attributable to members of the apolipoprotein B mRNA editing enzyme catalytic subunit‐like protein 3 (APOBEC3) family of single‐stranded DNA cytosine deaminases.^17^, ^18^, ^19^, ^20^ The APOBEC3 mutation signature is defined by C‐to‐T/−G single base substitutions in preferential 5′‐TCA and 5′‐TCT trinucleotide motifs (single base substitution signatures SBS2 and SBS13, respectively).^19^, ^20^ The APOBEC3 mutation signatures dominate a multitude of cancer types including those of the head/neck, cervix, bladder, breast, and lung.^17^, ^18^, ^19^, ^20^, ^21^, ^22^, ^23^ There are several lines of evidence in cancer biology indicating that APOBEC3 enzymes drive tumour evolution by promoting resistance to administered therapeutic regimens, aggressive subclonal expansion, and, thus, poor clinical outcomes.^24^, ^25^, ^26^, ^27^

The human APOBEC3 family comprises seven enzymes, A3A‐D and A3F‐H, that function to provide innate immune protection from infection by retroviruses (e.g. HIV‐1), herpesviruses (e.g. EBV), and human papillomaviruses (e.g. HPV16).^28^, ^29^, ^30^ Among the APOBEC3 enzymes, APOBEC3A (A3A) and APOBEC3B (A3B) are the most likely sources of the overall APOBEC‐driven mutations in human tumours.^20^, ^21^, ^31^ Large‐scale genomic analyses have revealed substantially elevated *A3B* expression levels and clear evidence for kataegis events in cutaneous melanomas,^21^ but low proportions of APOBEC3 mutation signatures SBS2 and SBS13.^9^, ^20^ However, skin melanomas also feature a strong dipyrimidine‐focused C‐to‐T mutation pattern that could eclipse an A3B deamination signature.^21^ In this context, A3B may provide insight into the pathogenesis of head/neck MMs that occur with minimal UV exposure. To address this hypothesis, we implemented immunohistochemistry approaches utilising our newly developed rabbit anti‐human A3B monoclonal antibody (mAb),^32^ in combination with next‐generation sequencing methods and analysis of available genomic datasets.

## Methods

### Case selection and tissue procurement

Following Institutional Review Board approval, formalin‐fixed paraffin‐embedded (FFPE) archival tissue blocks of primary oral MMs (*n* = 13) and oral melanocytic nevi (*n* = 13) were obtained from the Oral Pathology Laboratories of the School of Dentistry, University of Minnesota, and University of Florida College of Dentistry. In addition, primary sinonasal MMs (*n* = 13) were procured from the Department of Laboratory Medicine and Pathology, Medical School, University of Minnesota. All human tissues were derived from incisionally or excisionally biopsied head/neck mucosal lesions diagnosed between 2005 and 2018, and were classified according to the criteria of the most recent WHO Classification Head and Neck Tumours^1^ and Skin Tumours.^2^ Cases with a previous history of skin melanoma or MM of other anatomic sites were excluded. Haematoxylin and eosin (H&E)‐stained slides were reviewed to confirm the diagnoses and to assess the cytologic and histomorphologic features of each specimen, including tumour growth pattern and predominant cell phenotype. The epidemiologic characteristics of each patient (age and gender) and anatomic location of lesions were retrieved and tabulated (Tables 1 and 2).

**Table 1:** Presentation of the epidemiologic, histopathologic, and APOBEC3B (A3B) immunophenotypic characteristics of the primary head and neck mucosal melanomas included in this cohort

Case #	Gender/age (years)	Location	Growth pattern	Ulceration	Cellular features	Mitoses per mm^2^	TILs	Necrosis	PNI	LVI	APOBEC3B staining (H‐score)	Ancillary IHC markers
Oral mucosal melanomas												
1M	M/54	Palate	Nodular	Absent	Epithelioid, spindle	1	Non‐brisk	Absent	No	No	Selective, nuclear (26)	S100^+^, HMB45^+^
2M	F/68	Floor of mouth	Nodular	Absent	Spindle	0	Non‐brisk	Absent	No	No	Selective, nuclear (23)	Not available
3M	M/69	Palate/ maxillary gingiva	Nodular	Present	Epithelioid, spindle	2	Non‐brisk	Absent	No	No	Selective, nuclear (40)	S100^+^, HMB45^+^, Melan A^+^, Tyrosinase^+^
4M	M/71	Maxillary gingiva	Nodular	Present	Spindle	1	Non‐brisk	Absent	No	No	Diffuse, nuclear (43)	Melan A^+^
5M	F/71	Palate	Nodular	Present	Epithelioid	14	Non‐brisk	Absent	No	No	Diffuse, nuclear (46)	S100^+^, HMB45^+^
6M	M/62	Palate	Melanoma in situ with invasion	Absent	Epithelioid	3	Brisk	Absent	No	No	Selective, nuclear (37)	S100^+^, HMB45^+^, Melan A^+^
7M	F/62	Palate	Nodular	Present	Epithelioid, spindle	17	Non‐brisk	Absent	No	No	Negative (9)	Not available
8M	F/79	Maxillary gingiva	Nodular	Present	Spindle	50	Non‐brisk	Absent	No	No	Diffuse, nuclear (46)	S100^+^, HMB45^+^, Melan A^+^, Tyrosinase^+^
9M	M/69	Palate	Melanoma in situ with invasion	Absent	Epithelioid	0	Non‐brisk	Absent	No	No	Selective, nuclear (37)	HMB45^+^, Melan A^+^
10M	M/76	Palate	Nodular	Present	Spindle	1	Non‐brisk	Absent	No	No	Selective, nuclear (66)	S100^+^, HMB45^+^, Melan A^+^, Tyrosinase^+^
11M	F/71	Mid‐palate, torus area	Nodular	Absent	Spindle, epithelioid	4	Brisk	Absent	No	No	Diffuse, nuclear (53)	S100^+^, HMB45^+^, Melan A^+^
12M	F/46	Maxillary tuberosity	Nodular	Present	Spindle, epithelioid	10	Non‐brisk	Present	No	Yes	Diffuse, nuclear (72)	SOX10^+^, Melan A^+^, HMB45^+^
13M	F/89	Maxillary tuberosity	Nodular	Present	Spindle	6	Non‐brisk	Absent	No	No	Selective, nuclear (26)	SOX10^+^, S100^+^, Melan A^+^, HMB45^+^
Sinonasal mucosal melanomas												
14 M	F/71	Maxillary sinus	Nodular	Absent	Epithelioid, plasmacytoid	4	Non‐brisk	Present	No	No	Diffuse, nuclear (78)	HMB‐45^+^, Melan A^+^
15 M	F/89	Maxillary sinus	Nodular	Absent	Epithelioid, spindle	18	Non‐brisk	Absent	No	No	Diffuse, nuclear (99)	Negative for melanocytic markers
16 M	F/53	Pharynx	Nodular	Absent	Epithelioid, rhabdoid, multinucleated, anaplastic	21	Non‐brisk	Present	No	No	Diffuse, nuclear (54)	S100^+^, HMB45^+^, Tyrosinase^+^, SOX10^+^
17 M	M/64	Ethmoid	Nodular	Absent	Epithelioid, spindle	16	Non‐brisk	Absent	No	No	Negative (6)	S100^+^, HMB45^+^, Melan A^+^, Tyrosinase^+^
18 M	F/84	Nasal cavity	Nodular	Absent	Epithelioid, plasmacytoid	25	Non‐brisk	Absent	Yes	No	Diffuse, nuclear (54)	S100^+^, Melan A^+^
19 M	F/66	Nasal cavity	Nodular	Present	Epithelioid	25	Non‐brisk	Absent	No	No	Negative (9)	Not Available
20 M	F/69	Frontal sinus recess	Nodular	Present	Epithelioid, plasmacytoid, rhabdoid	5	Non‐brisk	Present	No	No	Selective, nuclear (24)	S100^+^, HMB45^+^
21 M	F/70	Nasal cavity	Nodular, peritheliomatous	Present	Epithelioid	6	Non‐brisk	Present	No	No	Negative (1)	S100^+^, HMB45^+^, Melan A^+^
22 M	M/91	Sinonasal, NOS	Nodular	Present	Epithelioid, plasmacytoid, anaplastic	4	Non‐brisk	Present	No	No	Selective, nuclear (15)	S100^+^, HMB45^+^, Melan A^+^, SOX10^+^
23 M	M/64	Nasal cavity	Nodular, peritheliomatous	Present	Epithelioid, plasmacytoid	10	Non‐brisk	Absent	Yes	No	Diffuse, nuclear (40)	S100^+^, HMB45^+^, Melan A^+^
24 M	F/67	Sinonasal, NOS	Nodular	Absent	Epithelioid, spindle, rhabdoid, multinucleated, anaplastic	18	Non‐brisk	Absent	No	No	Diffuse, nuclear (110)	SOX10^+^, S100^+^
25 M	M/73	Maxillary sinus	Nodular	Present	Epithelioid	10	Non‐brisk	Present	Yes	No	Negative for A3B (6); strong and diffuse, cytoplasmic A3G staining	S100^+^, HMB45^+^, Melan A^+^, SOX10^+^
26 M	M/51	Nasal cavity	Nodular	Present	Epithelioid, spindle	5	Non‐brisk	Absent	Yes	No	Negative for A3B (7); strong and diffuse, cytoplasmic A3G staining	S100^+^, HMB45^+^, Melan A^+^, SOX10^+^

M, male; F, female; TILS, tumour‐infiltrating lymphocytes; PNI, perineural invasion; LVI, lymphovascular invasion; IHC, immunohistochemistry; A3G, APOBEC3G; NOS, not otherwise specified.

**Table 2:** Presentation of the epidemiologic, histopathologic and APOBEC3B (A3B) immunohistochemical characteristics of the intraoral nevi

Case number	Gender	Age (years)	Location	Histopathologic subtype	APOBEC3B staining (H‐score)
1N	Male	30	Buccal mucosa	Intramucosal	Negative (4)
2N	Female	36	Buccal mucosa	Compound	Negative (3)
3N	Female	39	Buccal mucosa	Compound	Negative (2)
4N	Male	36	Buccal mucosa	Intramucosal	Negative (8)
5N	Male	34	Buccal mucosa	Intramucosal	Negative (6)
6N	Female	73	Gingiva	Intramucosal	Negative (4)
7N	Female	49	Palate	Intramucosal	Negative (7)
8N	Female	59	Gingiva	Intramucosal	Negative (3)
9N	Male	61	Buccal mucosa	Intramucosal	Negative (5)
10N	Male	84	Gingiva	Intramucosal	Negative (8)
11N	Male	29	Gingiva	Intramucosal	Negative (3)
12N	Male	27	Palate	Intramucosal	Negative (1)
13N	Male	69	Palate	Intramucosal	Negative (5)

### Immunohistochemistry (IHC)

The complete panel of primary antibodies against APOBEC3 proteins and ancillary IHC melanoma markers along with information regarding the clone, dilution, incubation time, and antigen retrieval methods were tabulated (Table S1). IHC staining for APOBEC3 proteins was performed following a previously described protocol.^32^, ^33^, ^34^ All melanoma marker stains were performed on a Ventana NexES automated system (Ventana Medical Systems, Tucson, AZ, USA) according to the manufacturers' instructions with appropriate positive and negative controls.

### A3B IHC quantification and statistical analyses

Nuclear A3B immunostaining was visualised with the Aperio ScanScope XT (Leica Biosystems, Wetzlar, Germany) and quantified using the Aperio Nuclear Algorithm software, as previously.^32^, ^33^, ^34^ For each case, the entirety of the stained tumour was annotated for analysis and calculation of corresponding A3B histoscore (H‐score).^35^, ^36^ Adjacent normal structures i.e. surface epithelium, were excluded from this analysis. Tumours with an H‐score ≤10 were considered negative for A3B. Since data were not distributed normally, statistical differences between groups were calculated using Kruskal–Wallis one‐way nonparametric tests and median and interquartile ranges were reported. *P* < 0.05 was considered statistically significant.

### RNA extraction and *APOBEC3* mRNA quantitative polymerase chain reaction (RT‐PCR)

Four 10 μm‐thick sections were collected from FFPE tissue blocks, deparaffinised in xylene and lysed in ice‐cold RLT and BME buffer according to the Qiagen (Chatsworth, CA, USA) cell lysis protocol. RNA isolation, cDNA synthesis and qPCR were performed as previously described.^18^ Experiments were performed in triplicate. The mean and standard error of the mean (SEM) of at least three independent experiments is presented.

### Next‐generation sequencing (NGS)

Genomic DNA was extracted from the FFPE samples and enriched DNA libraries were prepared and sequenced on an Illumina MiSeq instrument (v. 3 chemistry, 2 × 300 PE; Illumina, San Diego, CA, USA). FASTQ files were processed through a custom‐designed bioinformatics pipeline as described.^37^, ^38^ Amplicons with <500× minimum coverage were flagged for limited analytic performance. Additionally, variant call files (vcf) were filtered to remove calls with variant allele fractions (VAF) outside of the thresholds defined for accepted single nucleotide variants (5–10%) and insertion/deletion variants (1–5%). Clinically relevant mutations from this filtered variant list were annotated using the GenomOncology (Cleveland, OH, USA) software and reported. The analytic accuracy of the software is 99%. The panel of studied genes, which varied depending on the case, as well as sequenced exons were identified below: *BRAF* (NM_004333.4): exons 11, 12, 14, 15; *GNA11* (NM_002067.2): exon 5; *GNAQ* (NM_002072.3): exon 5; *KIT* (NM_000222.2): exons 2, 8‐14, 17, 18; *MAP2K1* (NM_002755.3): exons 2, 3, 6, 7; *NRAS* (NM_002524.4): exons 2‐4; *HRAS* (NM_005343.2): exons 2‐3; *PDGFRA* (NM_006206.4): exons 12, 14, 15, 18.

### Analysis of mucosal melanoma whole‐genome sequencing (WGS) data

WGS datasets for all available MM tumour samples (*n* = 8) in the International Cancer Genome Consortium (ICGC) were downloaded from the ICGC data portal (https://dcc.icgc.org/). Importantly, these specimens, originally reported by Hayward *et al*.,^9^ were analysed previously for the contribution of mutational signatures 2 and 13, but were reanalysed here using the APOBEC3 enrichment score as an improved metric for identifying APOBEC3‐specific mutations in these tumours. Only single base substitution (SBS) mutations were used to calculate the total number of mutations and total number of C‐to‐T mutations in this analysis (i.e. INDELs and other more complex somatic variations were filtered out). The APOBEC3 enrichment score was calculated as described.^39^, ^40^ In addition, we calculated an A3B‐specific enrichment score that only considers C‐T/G mutations in an (A/G)TCW context, which is the context preferentially selected by A3B. The identification numbers, available clinical information, and APOBEC3 and A3B‐specific enrichment scores of the ICGC cases are provided as Table S2.

## Results

### Clinicopathologic features of primary head/neck MMs and intraoral melanocytic nevi

The epidemiologic, clinicopathologic, and immunohistochemical characteristics of the 26 cases of primary head/neck MM are highlighted in Table 1. Fifteen cases affected women and eleven men, with a mean age at diagnosis of 69.2 years (age range = 51–91 years). Among the 13 intraoral MMs, palate was the most common site of involvement (7 of 13, 54%) followed by the maxillary gingiva (3 of 13, 23%) and maxillary tuberosity (2 of 13, 15%). The nasal cavity (5 of 13, 38%) and maxillary sinus (3 of 13, 23%) comprised the most frequent locations for primary sinonasal MMs.

**Figure 1:**
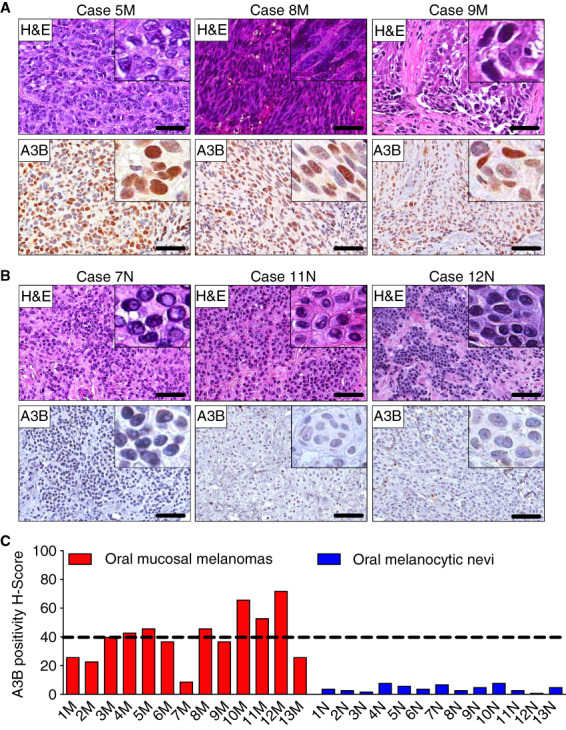
Endogenous A3B protein expression in primary oral mucosal melanomas (MMs) and oral melanocytic nevi. (**A**) A3B staining and corresponding H&E‐stained photomicrographs of representative primary oral MMs. Scale bars are 60 μm and inset images are magnified 8‐fold. (**B**) A3B immunophenotype and corresponding H&E‐stained photomicrographs of representative benign oral melanocytic nevi. Scale bars are 60 μm and inset images are magnified 8‐fold. (**C**) Collective presentation of quantified A3B IHC score (H‐score) of oral MMs (*n* = 13) and oral nevi (*n* = 13, control). The black horizontal dotted line indicates the A3B H‐score median (40) of the oral MMs group. [Color figure can be viewed at wileyonlinelibrary.com]

**Figure 2:**
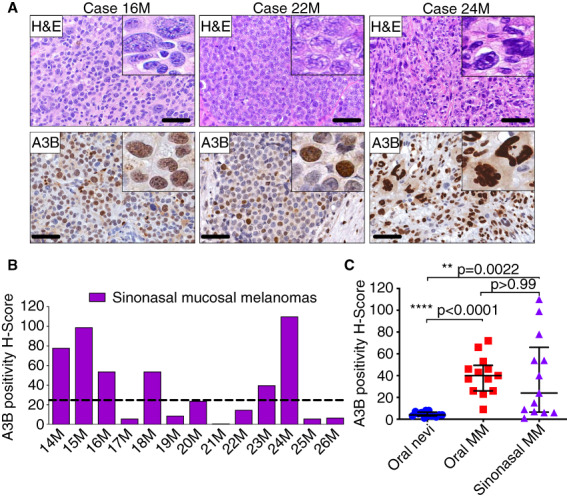
Endogenous A3B protein expression in primary sinonasal MMs. (**A**) A3B staining and corresponding H&E‐stained photomicrographs of representative primary sinonasal MMs. Scale bars are 60 μm and inset images are magnified 8‐fold. (**B**) Collective presentation of quantified A3B H‐scores of sinonasal MMs (*n* = 13). The black horizontal dotted line indicates the A3B H‐score median (24) of this group. (**C**) Collective presentation and comparison of quantified A3B H‐scores of oral melanocytic nevi (*n* = 13), oral MMs (*n* = 13) and sinonasal MMs (*n* = 13). The H‐score median and interquartile range for each group are shown and statistical significance for key comparisons is indicated (Kruskal–Wallis test). [Color figure can be viewed at wileyonlinelibrary.com]

**Figure 3:**
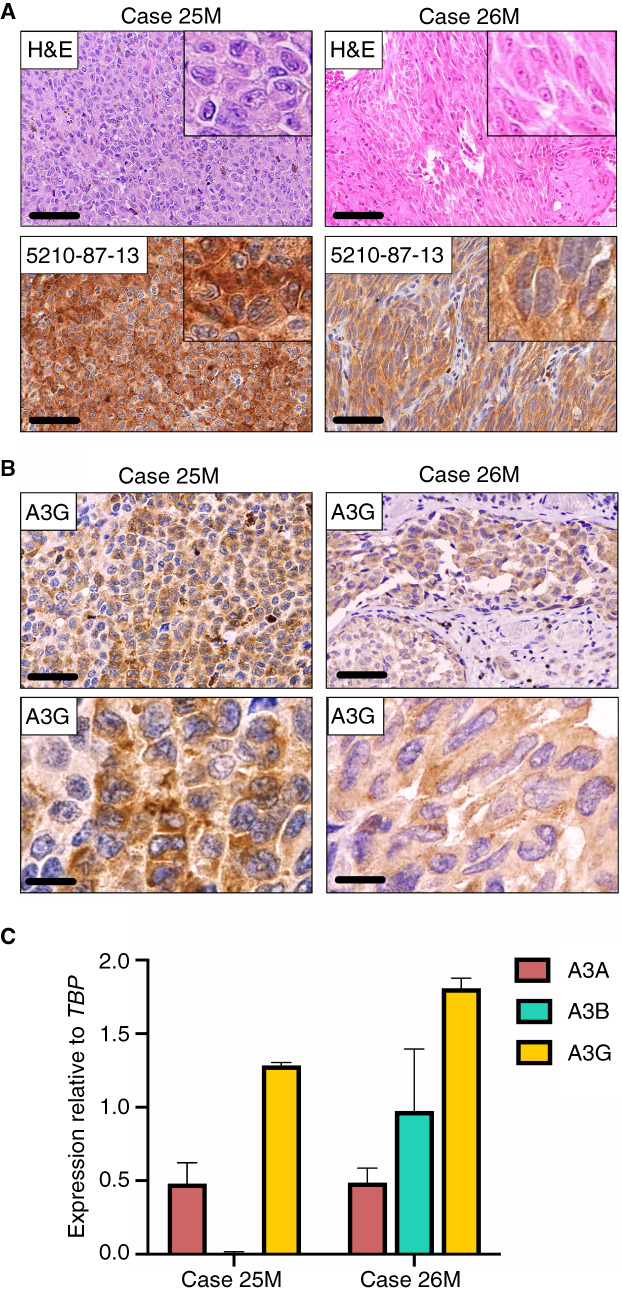
Endogenous A3G expression in primary sinonasal MMs. (**A**) H&E‐stained and corresponding 5210‐87‐13 immunohistochemical photomicrographs of the two cases with pronounced cytoplasmic positivity. Scale bars are 60 μm and inset images are magnified 8‐fold. (**B**) Staining with a commercial A3G specific mAb corroborates the presence of cytoplasmic A3G in these two sinonasal MMs. Scale bars are 60 and 20 μm, respectively. (**C**) Quantitative RT‐PCR investigating mRNA levels of *A3A*, *A3B*, and *A3G* expression in these two cases. Experiments were performed in triplicate. The mean and SEM of at least three independent experiments is shown. [Color figure can be viewed at wileyonlinelibrary.com] [Color figure can be viewed at wileyonlinelibrary.com]

Microscopically, 24 of 26 head/neck MMs (92%) exhibited a nodular growth pattern (Figures 1A and 2A) with a peritheliomatous (perivascular) component present in two of these cases. The remaining two MMs (8%) featured melanoma *in situ* with areas of invasion (Figure 1A; case 9M). Epithelioid cellular morphology was seen in 21 of 26 (81%) cases (Figure 1A; cases 5M and 9M), while a spindle cell phenotype was appreciated in 14 of 26 (54%) head/neck MMs (Figure 1A; case 8M, Figure 3A; case 26M). Frequently, a combination of epithelioid and spindle neoplastic cells was observed (9 of 26, 35%). Less common morphologic characteristics were observed in 7 of 26 (27%) lesions and included plasmacytoid, rhabdoid or highly anaplastic cells, and multinucleation (Figure 2A). Surface ulceration and necrosis were evident in 15 of 26 (58%) and 7 of 26 (27%) tumours, respectively (Table 1). Most lesions showed an increased number of mitoses, including atypical mitotic figures, that ranged from 0 to 50 per mm^2^ (mean = 11). Perineural and lymphovascular invasion were, overall, infrequent and seen in 4 of 26 (15%) and 1 of 26 (4%) head/neck MMs, respectively. Tumour infiltration by lymphocytes was predominantly focal (nonbrisk; 24 of 26, 92%) and rarely brisk (2 of 26, 8%).

The panel of ancillary IHC markers most frequently utilised to confirm the diagnosis of MM comprised S100, HMB45, tyrosinase, melan‐A (MART1), and SOX10 (Table 1). Eighteen cases stained for S100 were positive, showing strong and diffuse, cytoplasmic, and/or nuclear immunoreactivity. Furthermore, 19 and 17 head/neck MMs displayed strong cytoplasmic immunoreactivity for HMB45 and melan‐A, respectively. Finally, seven cases were stained for SOX10 and showed strong and diffuse, nuclear positivity. One case was negative for all melanocytic markers reported above.

The epidemiologic and clinicopathologic features of the 13 intraoral melanocytic nevi (control group) are summarised in Table 2. Eight cases affected men and five women with a mean age of 48 years (age range = 27–84 years). Buccal mucosa was the most frequent site (6 of 13, 46%) followed by the gingiva (4 of 13, 31%) and palate (3 of 13, 23%). Histopathologically, 11 of 13 (85%) nevi were classified as intramucosal and 2 of 13 (15%) as compound. The melanocytic nevi were composed of aggregates of ovoid or epithelioid nevus cells with abundant cytoplasm and no evidence of cytologic atypia (Figure 2B).

### A3B overexpression characterizes primary oral MMs but not benign intraoral nevi

With the exception of one case (7.7%), all oral MMs studied (12 of 13, 92.3%) showed heterogeneous, selective‐to‐diffuse, nuclear only, A3B immunopositivity in the majority of neoplastic cells (H‐score range = 9–72, median = 40; Figure 1A). A3B expression was seen in both epithelioid and spindle melanoma cells with varying staining intensity (Figure 1A, inset images). In contrast, benign oral melanocytic nevi (*n* = 13) were consistently and uniformly negative for A3B (H‐score range = 1–8, median = 4; Figure 1B). Collectively, primary oral MMs exhibited markedly elevated A3B IHC levels and corresponding H‐scores compared to melanocytic nevi (Figure 1C).

### A3B upregulation is a common finding in primary sinonasal MMs

Because A3B expression is evidently elevated in oral MMs, we then probed A3B protein levels by IHC in a group of sinonasal MMs and compared it to the intraoral tumours. Eight of 13 (62%) cases of sinonasal MM displayed heterogeneous A3B nuclear immunostaining (H‐score range = 1–110; median = 24; Figure 2B) that was mostly diffuse (six of eight, Figure 2A; cases 16M and 24M) and less frequently selective or rare (Figure 2A; case 22M). The A3B immunophenotypic properties of these cases are shown in Table 1.

A3B protein expression was increased significantly in oral and sinonasal MMs when compared to oral melanocytic nevi (*P* < 0.0001 and *P* = 0.0022, respectively, by Kruskal–Wallis test; Figure 2C). Notwithstanding notable intertumoral heterogeneity in the sinonasal MMs that is reflected in their corresponding A3B H‐score range, A3B levels were similarly elevated in oral and sinonasal tumours (*P* > 0.99 by Kruskal–Wallis test; Figure 2C).

Interestingly, two sinonasal MMs (cases 25M and 26M) stained with our 5210‐87‐13 mAb showed strong and diffuse cytoplasmic immunoreactivity (Figure 3A, inset images), but were negative for nuclear A3B (see Table 1). Since endogenous A3B exhibits exclusively nuclear localisation, the observed cytoplasmic staining pattern is consistent with A3A or A3G; both enzymes are also recognised by 5210‐87‐13 due to high homology of their C‐terminal domain.^32^ Staining of the lesions with a commercially available α‐A3G rabbit mAb (see Table S1) revealed the presence of cytoplasmic A3G in the epithelioid and spindle tumour cells (Figure 3B). Similar results were obtained upon analysis of *A3A*, *A3B*, and *A3G* mRNA levels by qRT‐PCR (Figure 3C). *A3G* mRNA expression was approximately 1.5‐ and 2‐fold higher in cases 25M and 26M, respectively, relative to the housekeeping gene *TBP*, whereas *A3A* and *A3B* were expressed at markedly low levels (Figure 3C).

Collectively, these data strongly indicate that A3B upregulation is a common underlying molecular event in head/neck MMs irrespective of the primary site, i.e. oral or sinonasal. Upregulation of other APOBEC members such as A3G can also occur (2 of 26, 8% of cases), but this phenotype is rare and appears to occur when A3B levels are low or absent.

### APOBEC3 enrichment scores are high in head/neck MMs but A3B does not drive *NRAS*, *HRAS,* or *KIT* genetic alterations in these tumours

**Figure 4:**
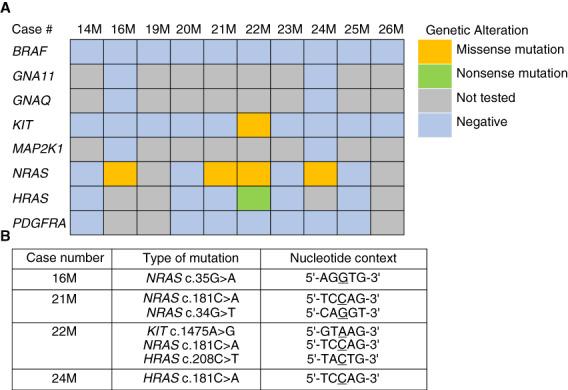
APOBEC3 does not drive *NRAS*, *HRAS*, or *KIT* genetic alterations in head/neck MMs. (**A**) NGS findings in primary sinonasal MMs (*n* = 10). (**B**) Type and nucleotide context of the most common pathogenic mutations discovered by NGS in primary sinonasal MMs. None of the *NRAS*, *HRAS*, or *KIT* base substitutions occurred in a 5′‐TCA‐3′ or 5′‐TCT‐3′ trinucleotide context, which represent the preferred DNA motifs for APOBEC3. [Color figure can be viewed at wileyonlinelibrary.com] [Color figure can be viewed at wileyonlinelibrary.com]

NGS was utilised to analyse the underlying oncogenic mutations in 10 primary sinonasal MMs. Four of eight cases (50%) tested harboured pathogenic missense *NRAS* mutations including p.Q61K, p.G12C, and p.G12D (Figure 4A). In addition, two variants of uncertain biologic significance were discovered in *KIT* (missense, p.K492R) and *HRAS* (nonsense, p.Q70*) as passenger mutations in a tumour (case 22M) with a concomitant *NRAS* mutation (Figure 4A). All 10 cases tested (100%) were negative for *BRAF* V600E alterations, which are common in other types of melanoma.^9^, ^41^ Notably, none of the above *NRAS*, *HRAS*, or *KIT* base substitutions occurred in 5′‐TCA‐3′ or 5′‐TCT‐3′ trinucleotide contexts, which represent preferred motifs for APOBEC3B‐catalysed deamination activity (Figure 4B).^21^, ^42^

**Figure 5:**
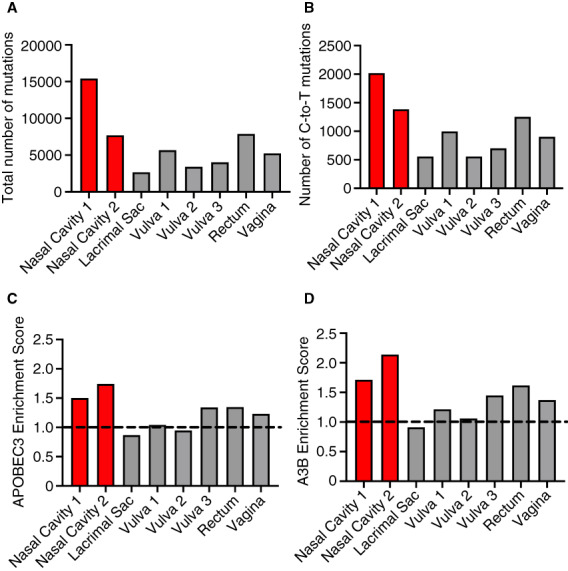
APOBEC3 enrichment scores are high in head/neck MMs. (**A**) Total number of mutations, (**B**) Number of C‐to‐T mutations, (**C**) APOBEC3 enrichment scores, and (**D**) A3B‐specific enrichment scores of eight cases of human MMs, including two nasal (red bars), available through public WGS databases. Enrichment score is a metric to represent the volume of APOBEC3‐specific single base substitutions T[C>T/G]W over background mutation at cytosine (at nonspecific contexts). A3B‐specific enrichment score exclusively considers C‐T/G mutations in an (A/G)TCW context, which is the context preferred by A3B. Red bars represent the two head/neck MMs that are significantly enriched for APOBEC3 and A3B mutations (*q* < 0.05). The dotted line at enrichment score = 1 is shown to visualize samples enriched (above 1) or depleted (below 1) for APOBEC3 or A3B mutations. These analyses remove C‐to‐A mutations. [Color figure can be viewed at wileyonlinelibrary.com] [Color figure can be viewed at wileyonlinelibrary.com]

Publicly available WGS data from eight human MMs arising in various anatomic sites were reanalysed, including two from the nasal cavity, which, as we showed above, are overall characterised by high A3B expression. The nasal MMs featured an increased total number of mutations (15,405 and 7701, respectively; Figure 5A), as well as a high number of C‐to‐T base substitutions (Figure 5B). The APOBEC3 enrichment score, an indicator of APOBEC3‐specific mutations, was markedly elevated in the two nasal cavity MMs when compared to all other mucosal tumours (Figure 5C, Table S2). Furthermore, when we examined C‐T/G mutations occurring in an (A/G)TCW context, the preferential context for A3B, the enrichment scores from the two nasal cavity MMs substantially increased (Figure 5D, Table S2). The latter strongly indicates a predominant role for A3B relative to other APOBEC3 enzymes, i.e. A3A, in generating the APOBEC mutational profile in those tumours.

## Discussion

The studies presented here are the first to investigate protein expression of the single‐stranded DNA cytosine deaminase A3B in primary head/neck MMs, a rare and particularly aggressive type of malignancy.^1^, ^3^, ^43^ IHC results here demonstrate elevated A3B protein levels in approximately 77% of the head/neck MMs studied. The A3B staining intensity and distribution showed inter‐ and intratumoral heterogeneity, a finding in agreement with A3B staining pattern in other human cancers, i.e. HPV‐positive and HPV‐negative head/neck squamous cell carcinoma^33^ and clear‐cell ovarian carcinoma^34^ that also exhibit increased, overall, A3B staining and the prevalence of APOBEC3 mutation signatures SBS2 and SBS13. Various molecular mechanisms participate in the regulation of A3B in human nonneoplastic tissues and cancers. A3B is induced by high‐risk HPV infections^44^, ^45^, ^46^ and directly regulated by the Rb/E2F cell cycle signalling pathway.^47^, ^48^, ^49^, ^50^, ^51^ Since there is no known causal association between viruses and MMs, it is plausible that A3B overexpression in head/neck MMs is driven by the pronounced proliferating properties of melanoma cells.

APOBEC3 deamination signatures (SBS2, SBS13) were found to dominate chromosomal regions with localised hypermutation (kataegis) in MMs and is associated with an increased number of structural rearrangements.^52^ APOBEC3 signatures were also present (≤30% contribution) in acral melanomas, which featured a higher number of gene rearrangements.^53^ Furthermore, SBS2 was the most common non‐UVR process identified in melanomas of adolescents and young adults.^54^ Only a low fraction of mutational signatures detected in adult cutaneous melanomas (1%) is attributable to APOBEC3.^9^, ^20^, ^54^ However, the prominent UVR‐related C‐to‐T mutation pattern (SBS7) present in skin melanomas could mask an A3B deamination signature.^21^ By revisiting publicly available WGS datasets we showed that both the APOBEC3 and A3B‐specific enrichment scores are significantly elevated in head/neck (nasal) MMs, despite the limited number of cases, and that A3B is not only overexpressed at the protein level, as indicated by IHC, but may also contribute to the overall heightened mutational burden in these tumours. Although the current study focuses mainly on head/neck MMs, high APOBEC3 enrichment scores were also observed in a few melanomas of other mucosae such as the vulva, rectum, and vagina.

Our studies herein have focused on A3B but do not exclude the possibility that other APOBEC3 enzymes such as A3A and A3H may also provide mutational fuel for head/neck MMs. To our knowledge, monoclonal antibodies have yet to be developed to specifically distinguish A3A and A3B. A3A and the catalytic domain of A3B are 92% identical at the protein level and the C‐terminal epitope recognised by our rabbit mAb 5210‐87‐13 is shared by these two enzymes.^32^ However, A3B is the only APOBEC3 family member that localizes constitutively to the nuclear compartment of cells,^32^, ^33^, ^34^, ^55^ whereas endogenous A3A is cytoplasmic in myeloid cell types and cell‐wide when overexpressed in heterologous systems.^56^, ^57^ In this study, only nuclear IHC staining was used to generate quantitative A3B H‐scores. Interestingly, two sinonasal MMs lacking nuclear A3B expression featured conspicuous A3G cytoplasmic staining. Although high A3G expression by tumour‐infiltrating T lymphocytes in the stroma of certain tumours, such as high‐grade serous ovarian carcinoma, correlates with improved outcomes,^58^ the biologic significance of A3G expression by the melanoma cells is currently unknown. Since A3G is confined exclusively to the cytoplasmic compartment of the cells,^55^, ^59^ it is not expected to contribute to the mutational load of these tumours.

Previous animal and cell line studies along with analysis of human datasets have shown that A3B overexpression drives drug resistance and tumour evolvability in various human malignancies, including oestrogen receptor‐positive breast tumours^24^ and EGFR‐ and ALK‐driven lung cancer.^27^ Conversely, depletion of A3B from cancer cells confers sensitivity and improved drug responses.^24^ Patients with head/neck MMs harbouring *KIT* mutations (approximately 25%) may benefit from treatment with imatinib.^3^ Notwithstanding promising initial responses, development of drug resistance is a frequent event in patients with MMs treated with targeted therapies against *KIT* mutations.^60^, ^61^ As we show here, A3B deaminating activity does not appear to relate with the pathogenic mutations in *KIT*, *HRAS*, or *NRAS* that characterize primary head/neck MMs. However, it is possible that A3B ongoing mutagenesis in these tumours may contribute to drug resistance. Unfortunately, no information on patient outcomes or clinical management was available in this cohort.

In conclusion, these novel immunohistochemical and genomic studies combine to strongly indicate a possible role for the single‐stranded DNA mutator A3B in primary mucosal melanomas of the head/neck, but not benign intraoral melanocytic nevi.

## Author contributions

P.P.A. performed study concept and design, provided acquisition, analysis, and interpretation of data, and statistical analysis, and prepared the initial draft of the article; J.N., M.C.J., P.E.W., and D.H. performed development of methodology and provided acquisition, analysis, and interpretation of data; M.N.I., I.B., R.G., F.L, and I.G.K. provided acquisition of data and material support; A.G. and R.S.H performed development of the methodology and writing, review and revision of the paper, and provided technical and material support. All authors read and approved the final paper.

## Conflict of interest

The authors have no conflicts to declare.

## Supporting information


**Table S1.** Primary antibodies utilised for the immunohistochemical characterisation of primary, oral and sinonasal, mucosal melanomas.

**Table S2.** Identification numbers, clinical information and APOBEC3 enrichment scores regarding the ICGC cases (*n* = 8) of human mucosal melanoma.

## Data Availability

The data that support the findings of this study are available from the corresponding author upon reasonable request.
