# Supplementary material for: Primary mucosal melanomas of the head and neck are characterised by overexpression of the DNA mutating enzyme APOBEC3B
Source: Histopathology. 2022 Dec 5;82(4):608–21. doi: 10.1111/his.14843 (PMC10107945; doi:10.1111/his.14843)
Supplement: Supplementary file 1 — Table S1. Primary antibodies utilised for the immunohistochemical characterisation of primary, oral and sinonasal, mucosal melanomas. [file HIS-82-608-s001.docx]

| **Antibody** | **Clone** | **Source** | **Dilution** | **Antigen retrieval method and time** | **Incubation period** |
| --- | --- | --- | --- | --- | --- |
| APOBEC3A/B/G | 5210-87-13,  rabbit monoclonal | Harris laboratory | 1:350 | Reveal Decloaker 35 min | Overnight at 4°C |
| APOBEC3G | HPA001812,  rabbit monoclonal | Sigma | 1:1,000 | Reveal Decloaker 35 min | Overnight at 4°C |
| S100 | rabbit polyclonal | Ventana | Predilute | CC1 36 min | 24 min |
| Melan A/ MART1 | A103 | Ventana | Predilute | CC1 64 min | 32 min |
| HMB45 | HMB-45 | Cell Marque | Predilute | CC1 64 min | 32 min |
| SOX10 | SP267 | Cell Marque | Predilute | CC1 64 min | 32 min |
| Tyrosinase | T311 | Ventana | Predilute | CC1 64 min | 16 min |

**Supplementary Table 1.** Primary antibodies utilized for the immunohistochemical characterization of primary, oral and sinonasal, mucosal melanomas.

CC1; cell conditioning 1
