# Supplementary material for: Primary mucosal melanomas of the head and neck are characterised by overexpression of the DNA mutating enzyme APOBEC3B
Source: Histopathology. 2022 Dec 5;82(4):608–21. doi: 10.1111/his.14843 (PMC10107945; doi:10.1111/his.14843)
Supplement: Supplementary file 2 — Table S2. Identification numbers, clinical information and APOBEC3 enrichment scores regarding the ICGC cases (n = 8) of human mucosal melanoma. [file HIS-82-608-s002.docx]

| **ID** | **Submitter ID** | **Sample ID** | **Gender** | **Specimen Type** | **Primary Site** | **APOBEC3 Enrichment Score** | **A3B-specific Enrichment Score** |
| --- | --- | --- | --- | --- | --- | --- | --- |
| DO220864 | MELA_0056 | EXTERN_MELA_20140514_026 | Male | Regional Lymph Nodes | Nasal Cavity 1 | 1.50 | 1.71 |
| DO222377 | MELA_0013 | EXTERN_MELA_20140505_023 | Female | Primary | Vulva 2 | 0.94 | 1.06 |
| DO222457 | MELA_0058 | EXTERN_MELA_20140514_030 | Female | Primary | Vulva 3 | 1.34 | 1.44 |
| DO222458 | MELA_0059 | EXTERN_MELA_20140514_032 | Male | Primary | Nasal Cavity 2 | 1.74 | 2.13 |
| DO222464 | MELA_0063 | EXTERN_MELA_20140514_041 | Female | Primary | Other - Lt lacrimal sac | 0.86 | 0.91 |
| DO222470 | MELA_0065 | EXTERN_MELA_20140514_045 | Female | Primary | Rectum | 1.34 | 1.61 |
| DO222481 | MELA_0071 | EXTERN_MELA_20140514_058 | Female | Primary | Vagina | 1.23 | 1.37 |
| DO222691 | MELA_0204 | EXTERN_MELA_20140528_006 | Female | Primary | Vulva 1 | 1.04 | 1.21 |

**Supplementary Table 2.** Identification numbers, clinical information and APOBEC3 enrichment scores regarding the ICGC cases (n = 8) of human mucosal melanoma.
